# Thermodynamic Analysis and Optimization of a High Temperature Triple Absorption Heat Transformer

**DOI:** 10.1155/2014/980452

**Published:** 2014-07-17

**Authors:** Mehrdad Khamooshi, Kiyan Parham, Mortaza Yari, Fuat Egelioglu, Hana Salati, Saeed Babadi

**Affiliations:** ^1^Department of Mechanical Engineering, Eastern Mediterranean University, G. Magosa, North Cyprus, Mersin 10, Turkey; ^2^Faculty of Engineering, Department of Mechanical Engineering, University of Mohaghegh Ardabili, Ardabil 179, Iran; ^3^Faculty of Mechanical Engineering, University of Tabriz, Tabriz, Iran; ^4^Department of Electrical and Computer Engineering, University of Concordia, Montreal, QC, Canada

## Abstract

First law of thermodynamics has been used to analyze and optimize inclusively the performance of a triple absorption heat transformer operating with LiBr/H_2_O as the working pair. A thermodynamic model was developed in EES (engineering equation solver) to estimate the performance of the system in terms of the most essential parameters. The assumed parameters are the temperature of the main components, weak and strong solutions, economizers' efficiencies, and bypass ratios. The whole cycle is optimized by EES software from the viewpoint of maximizing the COP via applying the direct search method. The optimization results showed that the COP of 0.2491 is reachable by the proposed cycle.

## 1. Introduction

Pollution, carbon dioxide production, environmental hazards, and global warming are the main disadvantages of fossil fuels. Alternative choices which can assist decision makers to avoid using fossil fuels have become a big challenge nowadays. Also huge amount of low or midlevel waste heat (60°C < *T* < 100°C) [1]. Absorption heat transformers which are capable of upgrading the energy efficiency of industrial applications appear to be a noble choice for utilizing these waste heats. They are systems that operate in a cycle opposite to that of absorption heat pumps (AHPs). Due to the fact that the fundamental of the AHTs is close to AHPs, absorption heat transformers will have the same advantages of the absorption systems such as quite operation, less maintenance requirement, less mechanical work input, and simple design [1–7].

The amount of gross temperature lift (GTL) basically depends on the additional stages added to the single absorption heat transformers (SAHTs).  Table 1 shows the coefficient of performance (COP) and GTL of the different types of absorption heat transformers.

The system performance of the SAHTs with different configurations using LiBr/H_2_O was presented by Horuz and Kurt [1]. They reported some enhancements in the COP of the SAHTs for modified configurations. Parham et al. [8] continued and optimized their work [1] and integrated them into a desalination system. They studied the effects of some major parameters such as heat source temperature, main components temperature, flow ratio on the performance of the systems, and fresh water production rate. Khamooshi et al. [9] conducted a similar research and coupled six different configurations of triple absorptions heat transformers into desalination systems. Zare et al. [10] proposed a new combined cogeneration system consisting of two organic Rankine cycles (ORCs) and a single absorption heat transformer. The required power for this system was provided by the waste heat of a gas turbine-modular helium reactor (GT-MHR). They demonstrated that the COP and the water production rate have direct relations with the heat source temperature of the AHT. Yari presented a novel cogeneration cycle comprised of a transcritical CO_2_ power cycle and a LiBr/H_2_O single absorption heat transformer [11]. The maximum water production rate of 3.317 kg/s was acquired by the cycle. Sözen and Yücesu [6] performed a mathematical simulation in order to compare the absorption heat transformers utilizing ejectors to the basic AHTs. Both cycles used H_2_O/NH_3_ as the working pair and employed the waste heat from a solar pond. The results demonstrated that both energy and exergy efficiency improvements were achievable by using ejector in the absorption heat transformer.

Siqueiros and Romero conducted two studies about recycling energy with and without increasing heat source temperature in AHT systems [12, 13]. They noticed some improvements in the proposed cycles than that of simple absorption heat transformer. Performance evaluation of the absorption heat transformers is possible by using artificial neural network analysis [14, 15]. Zhao et al. [16, 17] conducted a study to analyze three different configurations of double absorption heat transformers by the working pairs of LiBr/H_2_O. They concluded that the temperature of the absorbing evaporator is not an independent variable and mainly depends on the absorber temperature.

First and second law analysis of a double absorption heat transformer operating with LiBr/H_2_O were studied by Martínez and Rivera [18]. They formulated a mathematical model for estimating the COP, ECOP, total exergy destruction in the system, and exergy destruction in all the components as a function of temperature. Horuz and Kurt [19] investigated and compared single, series, and parallel double absorption heat transformers. They proved that the parallel double AHT system could generate more water vapor than that of the series double AHT system. A performance comparison of the single effect and double effect absorption heat transformer systems was made by Gomri [20]. The results showed that the double effect absorption heat transformers (DAHTs) had higher exergy and energy efficiencies and smaller water production than that of the single effect absorption heat transformers (SAHT). Also they discussed a solar absorption heat transformer coupled to a desalination system in a different study [21]. The proposed system could produce fresh water of 500 L per day in July for a beach house.

The performance of the absorption cycles depends not only on their configuration, but also on thermodynamic properties of working pairs which are regularly composed of refrigerants and absorbents [22]. A comparison was made between H_2_O/NH_3_ and LiBr/H_2_O solutions in single absorption heat transformer by Horuz [23]. Sun et al. [24] conducted a review study about different kinds of working pairs in absorption cycles. Furthermore, Khamooshi et al. [25] did a similar work for employed ionic liquids as working fluids. The same team published their second review paper wherein AHTs, from the view point of applications, crystallization risk, working fluids, performance evaluation, and economic aspects were reviewed comprehensively [26].

Donnellan et al. [27] introduced six different configurations of triple absorption heat transformers using LiBr/H_2_O as the working fluid. They optimized the number and locations of the heat exchangers within the system by reassembling them. In the second work, a rigorous multidimensional analysis was made for evaluating the performance of the triple absorption heat transformers by Donnellan et al. [28].

In this study a triple absorption heat transformer will be represented with a modified configuration described by Donnellan et al. [27]. However, their study did not investigate the effect of some major parameters on the performance of the system.

A thorough and comprehensive thermodynamic analysis will be performed for the best configuration of the latter mentioned study by the purpose of continuing it. A parametric study will be carried out and validated by other studies available from the literature in order to identify the effects of some parameters such as the temperatures of the condenser, evaporator, absorber, generator, first absorber/evaporator (AB/EV1) and second absorber/evaporator (AB/EV2), concentration of the weak and strong solution, and economizer effectiveness.

## 2. System Description

**Figure 1:**
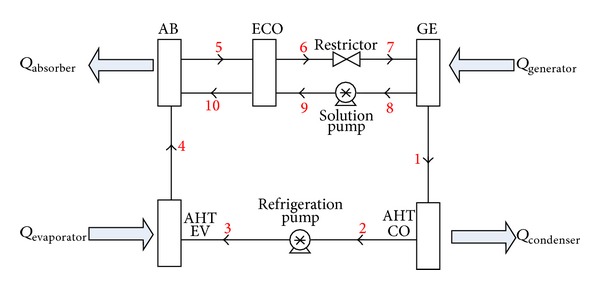
Schematic diagram of a single stage absorption heat transformer.

Figure 1 shows the general schematic of the absorption heat transformer in single stage mode. The SAHT basically consists of an evaporator, a condenser, a generator, an absorber, and a solution heat exchanger (SHE). The generator and evaporator are supplied with waste heat at the same temperature, leading to increased heat that can be collected at the absorber [26].

Refrigerant vapor is produced at state 4 in the evaporator by low or medium-grade heat source. The refrigerant vapor dissolves and reacts with the strong refrigerant-absorbent solution that enters the absorber from state 10, and the weak solution returns back to the generator at state 5. The heat released from the absorber will be higher than the input heat in generator and evaporator due to the exothermic reaction of LiBr and water in it. In the generator, some refrigerant vapor is removed from the weak solution to be sent to the condenser and, consequently, the strong solution from the generator is returned to the absorber. After condensing the vaporized refrigerant in the condenser, it is pumped to a higher pressure level as it enters the evaporator. The waste heat delivered to the evaporator causes its vaporization. Again, the absorber absorbs the refrigerant vapor at a higher temperature. Therefore, the absorption cycles have the capability of raising the temperature of the solution above the temperature of the waste heat [19].

**Figure 2:**
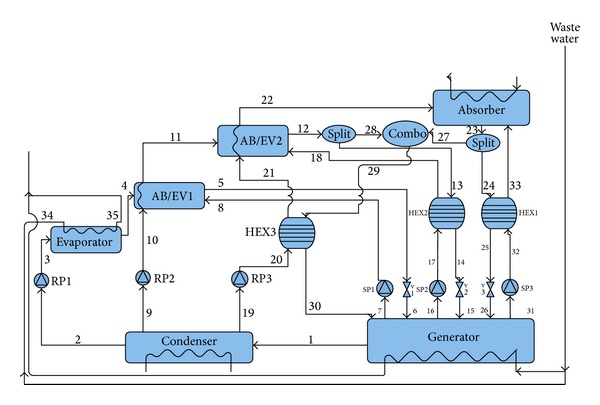
Schematic diagram of a triple absorption heat transformer.

Figure 2 displays the modified configuration of the triple absorption heat transformer. This cycle mainly consists of a generator, a condenser, an evaporator, an absorber, two absorber/evaporators, and three economizers. In this system heat is transferred to the evaporator and the generator from the waste hot water of a textile factory at the same time. The rejected heat from the absorber provides the thermal energy demanded by the desalination system. Superheated water vapor which works as refrigerant comes out from the generator and enters to the condenser where it is condensed as saturated liquid. One part of the condensed refrigerant is pumped to the higher pressure level of the evaporator (*P*_1_). In the evaporator, water is heated to saturated vapor phase with the same waste heat provided to the generator. This vapor is absorbed in the first absorber/evaporator by the strong solution of LiBr/H_2_O coming back from the generator. One portion of the released heat in the absorber is used to retain the absorber-evaporator-1 at a temperature higher than that of the evaporator. The second split of the condensed refrigerant leaving the condenser is pumped to the pressure level of* P*_2_ which is higher than that of* P*_1_ and provides heat to the saturated vapor by utilizing the heat of the absorption preserved by AB/EV1. This vapor is then absorbed in AB/EV2 by the strong LiBr/H_2_O solution flowing from the generator. Some parts of the absorption heat are used to maintain the AB/EV2 at a temperature higher than that of AB/EV1. The final split of the condensed refrigerant is pumped to the highest pressure level (*P*_3_) and consequently the water is heated in AB/EV2 to saturated vapor by the retained heat in the AB/EV2. Finally this saturated vapor is absorbed by the strong absorbent-refrigerant coming back from generator and the exothermic reaction in the absorber makes the temperature approximately (30–60°C [27]) hotter than that of the temperature of the AB/EV2. The residual part of the released heat is transferred to impure water as latent and sensible heat in desalination system as shown in Figure 2. The weak solutions coming back from the absorber and AB/EV2 are divided into two parts. One part of each is directly returned to generator and used in heat recovery part of the first and second heat exchanger. The second fractions of the weak solution are combined together in order to be used in heat recovery part of the third heat exchanger.

## 3. Thermodynamic Analysis

The following section describes the thermodynamics model used for simulation of the TAHT. Each component of the considered system has been treated as a control volume and the principles of mass and energy conservation are applied to them. EES software is used for solving the equations [32].

### 3.1. Assumption

The following assumptions have been made in the analysis.

1. Changes in kinetic and potential energies are neglected [8, 27, 28].
2. The pressure losses due to the frictional effects in the connecting pipes of the AHT are neglected [8, 18, 27, 28].
3. The system is in thermodynamic equilibrium and all the processes are assumed to be steady flow processes [8, 18, 27, 28].
4. Some proper values of effectiveness are considered for the heat exchangers [8, 18].
5. In the TAHT, the solution at the generator and the absorber outlets, as well as the refrigerant at the condenser and the evaporator outlets, is all at saturated states [8, 18, 27, 28].
6. The salt utilized in the absorbent solution is assumed to have negligible vapor pressure [27, 28].
7. Heat losses from components are very small relative to heat fluxes and are thus not included in the model [27, 28].
8. The evaporator and the generator of the AHT work at the same temperature [8].
9. The refrigerant vapor is assumed to evaporate and condense completely in the two absorber-evaporators, the evaporator, and the condenser [27, 28].
10. The heat source for the AHT system is the hot water generated by a cogeneration system in a textile company. The industrial system has four different units each of them producing 15 ton/h water at 90 ± 2°C [1, 8].
11. The mechanical energy consumed by pumps can be neglected [8].

### 3.2. Performance Evaluation

Table 2 summarizes the basic assumptions and used input parameters in the simulation.

The system's COP is determined as the ratio of useful heat output systems over the systems input energy. Due to the fact that COP is the most important criterion of the cycle's capability for upgrading the delivered thermal energy to the system, it plays an important role in analyzing the absorption cycles. The COP is given in the following equation [33–35]:

(1) $$\begin{array}{c} \text{COP}=\frac{Q_{\text{abs}}}{Q_{\text{gen}}+Q_{\text{eva}}}. \end{array}$$

Another fundamental parameter for designing and optimizing the absorption cycles is the flow ratio. It is defined as the ratio of the total mass flow rate of weak solution entering the generator to the mass flow rate of refrigerant vapour leaving the generator [27]:

(2) $$\begin{array}{c} f=\frac{\text{mass flow of salt solution entering the generator}}{\text{mass flow of vapour leaving the generator}}. \end{array}$$

The heat capacities of the main components of the cycles are as follow:

(3) Qeva=m˙34(h34−h35)=m˙4(h4−h3),Qgen=m˙1h1+m˙7h7+m˙16h16+m˙31h31−m˙6h6−m˙15h15−m˙30h30,Qcon=m˙1h1−m˙2h2−m˙9h9−m˙19h19,Qabs=m˙22h22+m˙33h33−m˙23h23,QAB/EV1=m˙10(h11−h10)=m˙4h4+m˙8h8−m˙5h5,QAB/EV2=m˙22(h22−h21)=m˙11h11+m˙18h18−m˙12h12.

The heat capacity of absorber also can be calculated by using the equation below [1, 19]:

(4) $$\begin{array}{c} q_{\text{abs}}=\frac{Q_{\text{abs}}}{{\dot{m}}_{4}}=\left(f+1\right)h_{23}-fh_{22}-h_{33}. \end{array}$$

Bypass ratio is an imperative parameter defined for the weak solutions coming back from the absorber and AB/EV2 defined as

(5) $$\begin{array}{c} \text{BP}1=\frac{m_{24}}{m_{27}},\\ \text{BP}2=\frac{m_{13}}{m_{28}}. \end{array}$$

### 3.3. Optimization Method

The cycle's performance fundamentally depends on temperature of the main components; therefore the optimum COP of the cycles can be expressed as follows:

(6) Maximize COP  (Tabs,Tgen,Tcon,TAB/EV1,TAB/EV2,Teva)Subject to:      20≤Tcon≤35C°             90≤Teva≤105C°             90≤Tgen≤105C°             110≤TAB1≤145C°             165≤TAB2≤180C°             180≤Tabs≤215C°.

By applying the constraint for each variable and by setting bounds, the performance of the whole cycle is optimized by EES software from the viewpoint of maximizing the COP.

Direct search method is best known as unconstrained optimization technique that does not explicitly use derivatives [36].

### 3.4. Model Validation

The available data in the literature were used to validate the simulation results. For the case of the AHT cycle the experimental results reported by Rivera et al. [31] are used. The conditions and assumptions used in their work are applied for the aim of validation. The assumptions are as follows.

- Heat losses and pressure drops in the connecting pipes and the components are considered negligible.
- The flow through the expansion valves is isenthalpic.
- The effectiveness of the economizer is 70%.
- The absorber temperature is 123°C.
- The generator and evaporator temperatures being the same are 74.1°C

**Figure 3:**
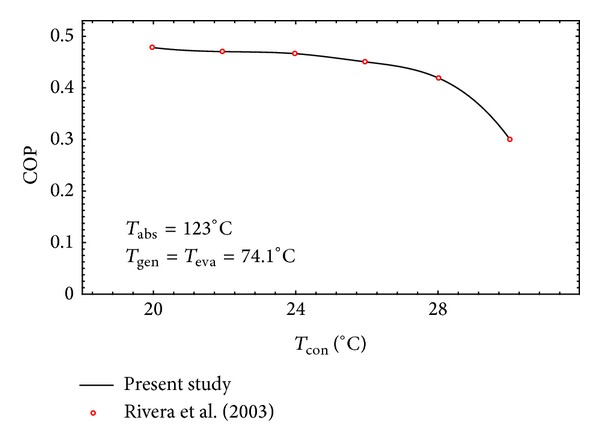
Validation of the simulation model developed for AHT system.

Figure 3 shows the comparison between the COP obtained from the present work with that reported by Rivera et al. [31] The figure shows an excellent agreement between the two and indicates a decrease in the COP as the condenser temperature of the AHT system increases.

**Figure 4:**
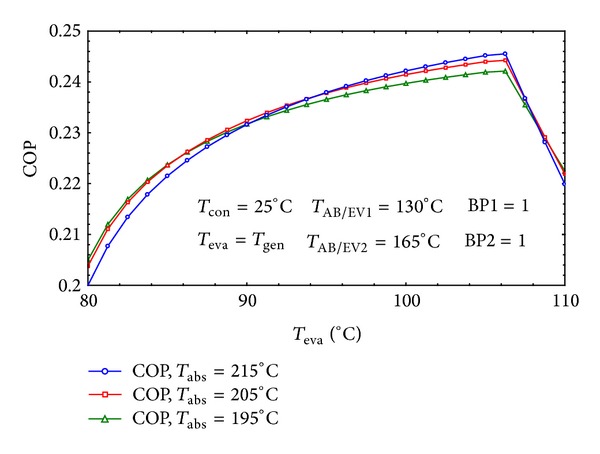
Effect of *T*_eva_ on the COP of the system.

**Figure 5:**
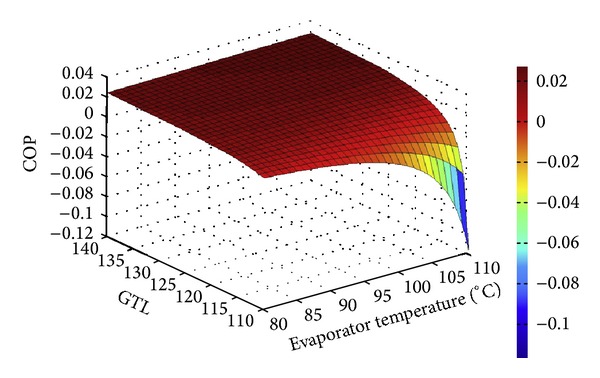
Effect of *T*_eva_ on the COP of the system [27].

The available result in the Donnellan et al.'s [27] work was also used to validate the simulation. The similarity between Figures 4 and 5 verifies the validity of our simulations. Likewise, the obtained COP of the system is qualitatively in agreement with the latter mentioned study.

In Figures 4 and 5 it is evident that as *T*_eva_ is higher than 105°C, COP drops and there is a satisfactory agreement between them.

## 4. Results and Discussion

**Figure 6:**
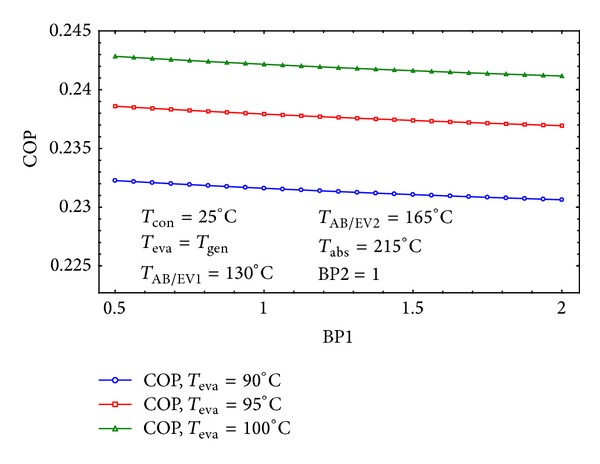
The effect of BP1 on the COP of the cycle for three different *T*_eva_.

**Figure 7:**
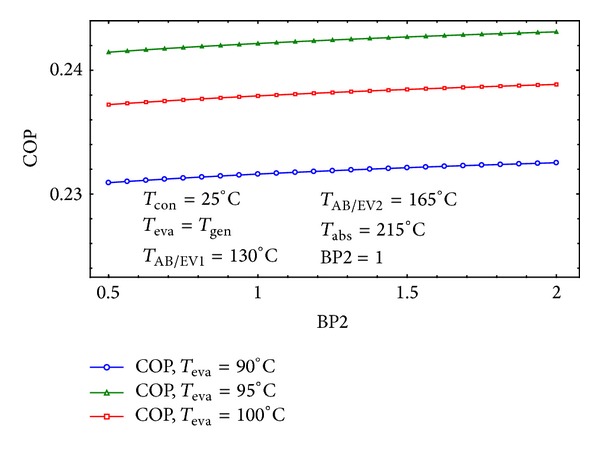
The effect of BP2 on the COP of the cycle for three different *T*_eva_.

Figures 6 and 7 show the variation of the COP by the first and second Bypass ratios. It is clear that the COP is higher for smaller amounts of BP1 and higher quantities of BP2. This means that the temperature of the point 29 will become higher with considered amounts of bypass ratios which leads to increasing heat recovery in the third heat exchanger. This is in agreement with those reported in [27] whom stated the fact that the third heat exchanger has a much greater influence on the cycle's performance than any other heat exchanger.

**Figure 8:**
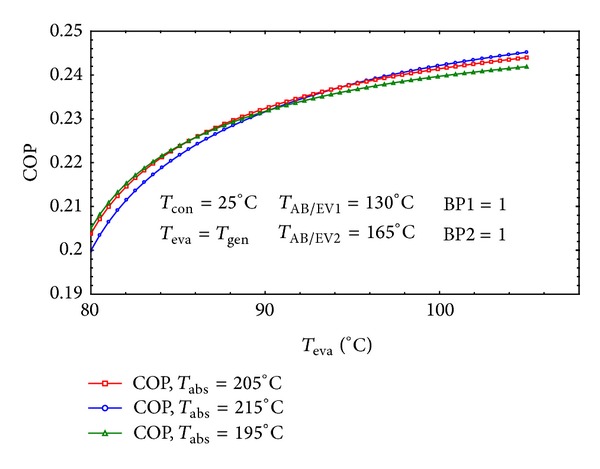
The effect of *T*_eva_ on the COP of the cycle for three different *T*_abs_.

As it can be seen in Figure 8 when *T*_eva_ which is equal to *T*_gen_ increases, the COP would also increase. This is due to the fact that the maximum pressure of the system has direct relation with the evaporator's temperature. By increasing the maximum pressure of the system the weak solution concentration will decrease by decreasing flow ratio. The lower flow ratio means higher absorber heat capacity and the higher COP. This result achieves harmony with those reported in [1, 10, 19, 27]. Also it is clear that as the gross temperature lift (GTL = *T*_abs_ − *T*_eva_) decreases the COP and increases rapidly. From the first law of thermodynamics it is explicit that the system has to reduce its efficiency as the absorber increases its temperature which is in accordance with the results available in [8, 27, 37, 38].

**Figure 9:**
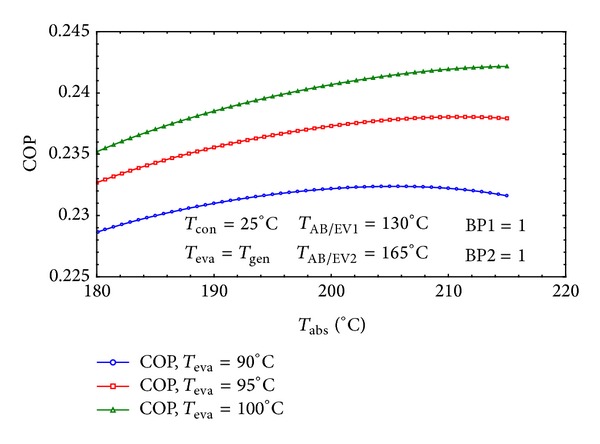
The effect of *T*_abs_ on the COP of the cycle for three different *T*_eva_.

Figure 9 depicts the variation of COP with *T*_abs_ in three different evaporator temperatures. It is clear that it has a similar trend with that of Figure 8 which shows the decreasing behavior of COP by increasing GTL. Also by increasing *T*_abs_, the concentration of the weak solution and consequently the flow ratio increase which leads into a decrease in the absorber heat capacity [8, 10, 20, 39].

**Figure 10:**
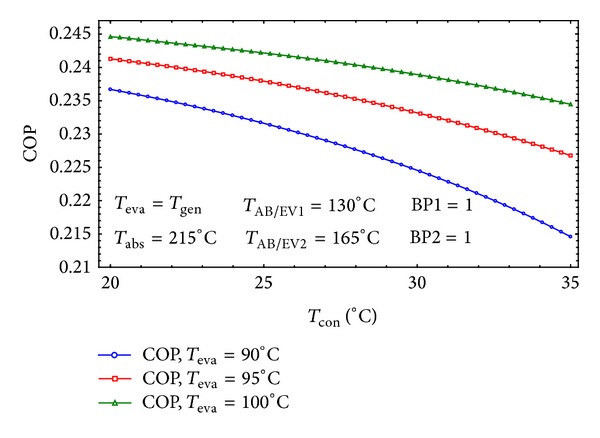
The effect of *T*_con_ on the COP of the cycle for three different *T*_eva_.

Figure 10 shows the effect of condenser's temperature on the COP of the system. This is by reason of the fact that the minimum system pressure raises with increasing the condenser temperature. By boosting the *T*_con_ the strong solution concentration will go down which increases the flow ratio of the system. The bigger flow ratio will result in the lower absorber heat capacity and lower COP [19]. Figure 10 proposes that the condensation temperature should always be kept at its lowest setting [28] and the system has its best performance during the winter.

Concentration of the working pair in the system can be classified into two categories: firstly, strong solution which is the concentration of the solution flowing from generator to absorber (*X*_31_) and secondly the weak solution that is the concentration of the solution coming back from the absorber to the generator (*X*_23_).

**Figure 11:**
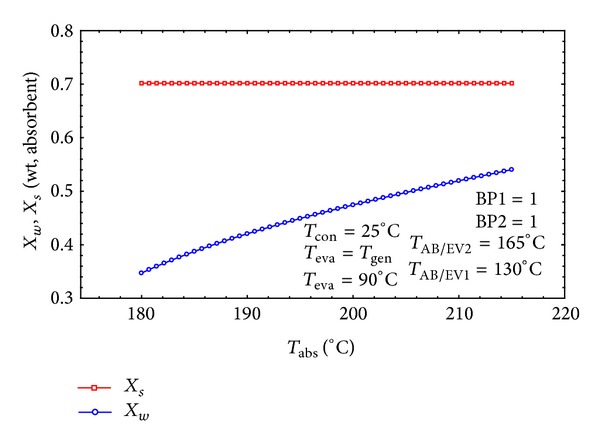
The effect of *T*_abs_ on the concentration of the strong and weak solution.

In Figure 11 both *X*_*s*_ and *X*_*w*_ are plotted against the absorber temperature. It is obvious that when generating, condensing, and absorber/evaporator temperatures are kept constant, *X*_*s*_ does not vary with *T*_abs_, but the *X*_*w*_ is increasing. The higher the evaporator or generator temperature is, the bigger the strong solution is. Consequently the higher *X*_*s*_ results in the higher flow ratio and can also increase the amount of the demanded heat input into the cycle [1].

**Figure 12:**
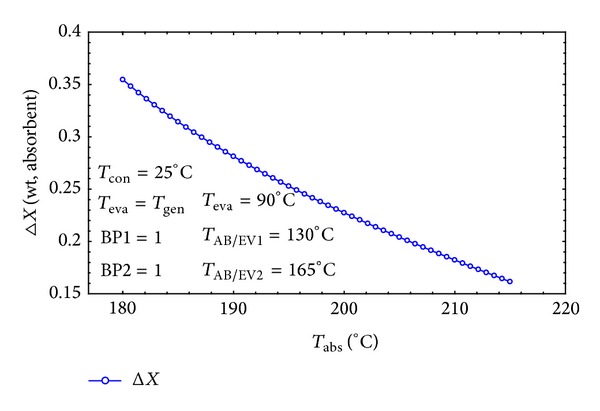
The effect of *T*_abs_ on Δ*x*.

The concentration difference (Δ*X* = *X*_*s*_ − *X*_*w*_) exhibits a parabolic decrease by increasing absorber temperature as shown in Figure 12. As mentioned correspondingly in the literature [8, 10, 40], when generation, evaporation, AB/EV, and condensing temperatures are constant, the Δ*X* and *T*_abs_ will only vary with *f*, which is an important and easily controllable operation parameter. Larger *f* also results in higher *T*_abs_ and more mechanical power losses. This is completely in agreement with the results of Horuz and Kurt [1] which proved that if the evaporator temperature is bigger, the AHT performs better.

**Figure 13:**
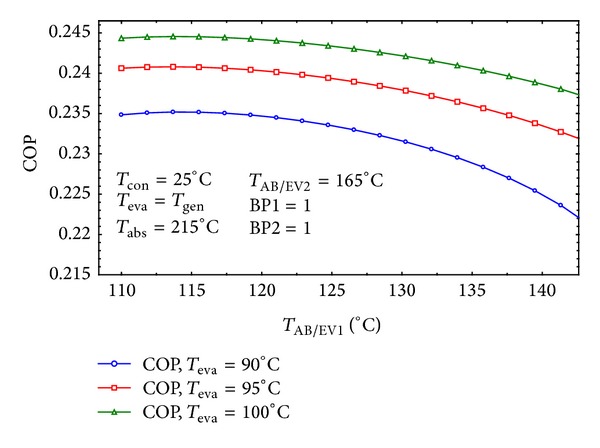
The effect of *T*_AB/EV1_ on COP of the system.

**Figure 14:**
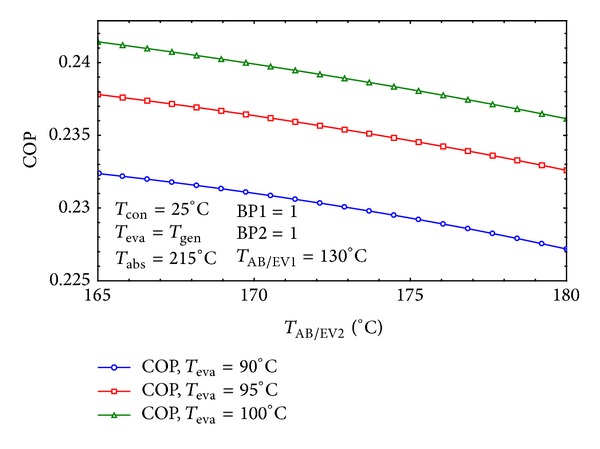
The effect of *T*_AB/EV2_ on COP of the system.

The effects of *T*_AB/EV1_ and *T*_AB/EV2_ on the COP of the system have been shown in Figures 13 and 14 for the considered settings. In Figure 13 the COP is at the highest value within the midpoint temperature of *T*_AB/EV1_. But as demonstrated in Figure 14 *T*_AB/EV2_ should remain as low as possible in order to achieve maximum COP.

**Figure 15:**
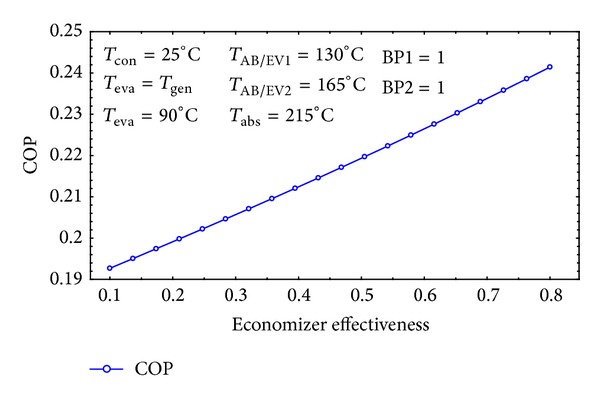
The effect of economizer on COP of the cycle.

In Figure 15 the COP is plotted against heat exchanger efficiencies (*ε*). It is obviously evident that *ε* has a major role on the COP of the system. The variation of the economizer effectiveness can increase the COP up to 21%. It can be concluded that the effectiveness of the economizers should be as high as possible, that is, a reasonable result according to the heat transfer phenomenon.

### 4.1. A General Comparison among Different Configurations of AHTs

**Figure 16:**
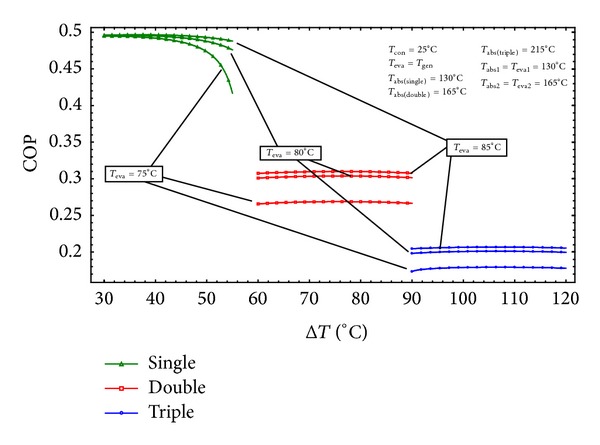
Effects of GTL on COP for different types of AHTs at different evaporation temperatures [26].

In our previous work [26] the variation of COP with gross temperature lift under different evaporation temperature conditions was compared for single, double, and triple AHTs, using LiBr/H_2_O the working fluid. As seen in Figure 16, any increase in GTL will cause a drop in the COP. This is due to the fact that as *T*_abs_ increases, the concentration of the weak solution and consequently the flow ratio (*f*) increases resulting in a decrease in the absorber heat capacity. This result is in agreement with that reported in the literature [1, 8].

Additionally, the higher the evaporation temperature is the higher the absorption temperature and the corresponding gross temperature are. It is observed that SAHT has the highest COP and the TAHT owns the lowest. The same trend has been reported by Donnellan et al. [27].

## 5. Optimization

By using direct search method in EES software, the COP magnitude of the cycle has been optimized as a function of main components' temperatures. The results are summarized in Table 3. The optimization was performed for 4 different evaporator temperatures.

The results are completely in coherence with the previously discussed results in the figures. The COP has ascending trend with increasing the temperature of the evaporator as earlier in Figure 8.

As shown in Table 3 the *T*_con_ is always at the lowest possible amount of the setting which is 20°C (discussed in Figure 10). Also the bypass ratios show a proper amount in comparison with Figures 6 and 7. The maximum amount of COP which is 0.2491 is obtained at the *T*_eva_ = 105°C.

## 6. Conclusion

In this study a triple absorption heat transformer is proposed for waste heat recovery from the industrial processes. Thermodynamic models were developed and parametric studies were carried out. Also performances of the whole cycle were optimized for maximum COP.

It is proven that in the triple absorption heat transformer systems,

- the systems condensation temperature should always be kept at minimum value;
- the lower gross temperature lift is, the higher the COP will be;
- the third heat exchanger has a much greater influence upon the cycle's performance than the any other heat exchanger;
- single stage absorption heat transformer has the highest COP and the TAHT owns the lowest;
- the maximum COP of 0.2491 is obtained from the cycle.

## Figures and Tables

**Table 1:** System performances of different types of absorption heat transformers.

Type	GTL	COP	Reference
Single absorption heat transformer (SAHT)	50°C	~0.5	[1, 8, 19]

Double absorption heat transformer (DAHT)	80°C	~0.35	[16–19]

Triple absorption heat transformer (THAT)	~140	~0.23	[27, 28]

**Table 2:** The input data of simulation.

Parameters	Value
*T*_con_ (°C)	20–35^a^
*T*_abs_ (°C)	180–215^b^
*T*_eva_ (°C)	80–110^c^
*T*_eva_ = *T*_gen_ (°C)	^ d^
*T*_AB/EV1_ (°C)	110–145^e^
*T*_AB/EV2_ (°C)	150–180^e^
*T*_heat source_(°C)	90 ± 2°C^f^
m-dot heat source (ton/h)	60^f^
*ε*_ECO_ (%)	80^g^

The values are obtained from various studies such as a [29–31], b [27, 28], c [27],d [8, 12, 13, 20, 21], e [27, 28], f [1, 8], g [8, 20, 21].

**Table 3:** The results of optimization for maximum value of the COP.

*T*_eva_	BP1	BP2	*T*_con_	*T*_gen_	*T*_AB/EV1_	*T*_AB/EV2_	*T*_abs_	COP
90	0.5	1.977	20	90	110	165	210.5	0.2423
95	0.5	2	20	95	111	165	211.2	0.2461
100	0.5	2	20	100	112.3	165	213	0.2487
105	0.5	2	20	105	115.4	165	215	0.2491
